# Implications of Persistent HPV52 and HPV58 Positivity for the Management of Cervical Lesions

**DOI:** 10.3389/fonc.2022.812076

**Published:** 2022-05-18

**Authors:** Baozhu Yi, Qian Xu, Zhixuan Zhang, Jinyi Zhang, Yi Xu, Luoqi Huang, Yue Hu, Quanmei Tu, Jingyun Chen

**Affiliations:** ^1^Department of Gynecology, Second Affiliated Hospital and Yuying Children's Hospital of Wenzhou Medical University, Wenzhou, China; ^2^Department of Gynecology, Yiwu Maternity and Children Health Care Hospital, Jinhua, China

**Keywords:** cervical lesions, human papillomavirus (HPV), colposcopic biopsy, human papillomavirus 52 (HPV52), human papillomavirus 58, cervical cancer

## Abstract

**Objective:**

This study aimed to compare the variability of HPV16/18/52/58 subtype infections in patients with different cervical lesions, to explore the guiding significance of persistent positive HPV subtypes 52 and 58 in the stratified management of cervical lesions, and to determine the appropriate management model.

**Method:**

This study was conducted through a retrospective analysis of 244,218 patients who underwent HPV testing at the Second Affiliated Hospital and Yuying Children’s Hospital of Wenzhou Medical University from September 2014 to December 2020 to examine the distribution of different types of HPV infection. From March 2015 to September 2017, 3,014 patients with known HPV underwent colposcopy to analyze high-risk HPV infection for different cervical lesions. Meanwhile, from September 2014 to December 2020, 1,616 patients positive for HPV16/18/52/58 alone with normal TCT who underwent colposcopy in our hospital were retrospectively analyzed for the occurrence of cervical and vulvovaginal lesions, with colposcopic biopsy pathology results serving as the gold standard for statistical analysis.

**Result:**

Analysis of 244,218 patients who had HPV tested revealed that the top 3 high-risk HPV types were HPV52, HPV58, and HPV16. Further analysis of 3,014 patients showed that 78.04% of patients referred for colposcopy had HPV16/18/52/58 alone. Among high-grade squamous intraepithelial lesions (HSIL) and cervical cancer, the most common is HPV16, followed by HPV58 and then HPV52 (*p* < 0.05). A total of 1,616 patients with normal TCT who were referred for colposcopy due to HPV16/18/52/58 infection were further analyzed. Based on pathological findings in lesions of HSIL and CC, HPV16 is the most common, followed by HPV58 and then HPV18 (*p* < 0.05). In the 1,616 patients analyzed, high-grade vulvovaginal lesions were detected, with HPV58 being the most common, followed by HPV16 and then HPV52 (*p* < 0.05).

**Conclusion:**

1. In patients with positive HPV58 alone and normal TCT, the indications for colposcopy may be relaxed, with particular attention paid to the possibility of vulvar and vaginal lesions.

2. Patients with a positive HPV type 52 alone and normal TCT may be considered for a follow-up review and, if necessary, a colposcopy.

3. The development of a more suitable HPV vaccine for the Asian population, such as HPV16/18/52/58, may better protect women’s health.

## Introduction

Cervical cancer is the 4th most common cancer among women (1) and is more prevalent in developing countries than in developed countries (2).

Persistent high-risk human papillomavirus (HR-HPV) infection with HPV is a major cause of cervical precancer and cervical cancer. By the time the HPV vaccine for cervical cancer became available in 2006, human research on the correlation between HPV and cervical disease had come a long way in just a few decades, with tremendous achievements in the understanding and study of HPV, with studies reported from all over the world. However, studies on cervical disease due to different subtypes of HPV infection have been reported inconsistently, and the interactions between the various subtypes of infection are inconsistent. More than 150 types of HPV have been identified, with more than 40 of which can cause cervical lesions. HPV testing is widely used to screen for cervical cancer and has significantly reduced the incidence and mortality rate of the disease.

This paper focuses on the analysis of four high-risk subtypes of HPV16/18/52/58 in different cervical lesions to understand the HPV infection in different cervical lesions. According to studies, the ranking of HR-HPV subtypes varies depending on the level of cervical lesions, with HPV52, HPV58, and HPV16 having the greatest impact on the health of Chinese women (3). HPV18, HPV16, HPV52, and HPV58 are more prevalent in patients with high-grade squamous intraepithelial lesions (HSIL) and invasive cervical cancer (4). HPV52 and HPV58 are more prevalent in squamous intraepithelial lesions and cervical cancer from East Asia than in other parts of the world (5). It has an important role in the development of cervical cancer in Chinese women (6). The implementation of stratified management of high-risk groups is important in reducing the incidence of cervical cancer and precancerous lesions, and both the ASCCP/ACS/ASCP and the CSCCP advocate immediate referral for colposcopy for those positive for HPV16/18 infection. However, there is uncertainty about the significance of HPV52 positivity and HPV58 positivity in the stratified management of cervical lesions. To investigate the guiding role of HPV52 and HPV58 in the stratified management of cervical lesions, this study was conducted. This study was conducted through a retrospective analysis of the patient population attending the gynecology department of our hospital and is reported below.

## Materials and Methods

### General Information

From September 2014 to December 2020, 244,218 patients aged 17–91 years, with a mean age of 39.9 ± 10.47 years, were tested for HPV at the Second Affiliated Hospital and Yuying Children’s Hospital of Wenzhou Medical University. Of the 244,218 patients, 1,616 patients aged 18–83 years, with a mean age of 41 ± 10.59 years, underwent colposcopy for HPV16/18/52/58 alone positive with normal TCT. Meanwhile, from March 2015 to September 2017, 3,014 patients with known HPV aged 17–82 years, with a mean age of 41.38 ± 10.67 years, underwent colposcopy.

### Method

#### HPV Testing

Cervical scrape specimens were gently collected from the squamocolumnar junction of the cervix using a sampling brush and were stored at 4°C prior to HPV genotyping. The genotype of HPV was determined using a 27-HPV genotyping kit from TellgenplexTMxMAP™ (TELLGEN Life Sciences Ltd., Shanghai, China), which detects 17 high-risk types (HPV16, HPV18, HPV26, HPV31, HPV33, HPV35, HPV39, HPV45, HPV51, HPV52, HPV53, HPV56, HPV58, HPV59, HPV66, HPV68, HPV82) according to the manufacturer’s instructions. Patients were grouped according to HPV16, HPV18, HPV52, and HPV58 infections. The HPV16/18/52/58 group indicates positivity for HPV species alone, whereas other groups include other types of infections or mixed infections.

#### Cervical Liquid-Based Cytology Tests

Cells were collected from the ectocervix and the cervical canal using a cervical canal brush, and the cells attached to the small brush were eluted in vials containing cell preservation solution and sent to the pathology department (Haoluojie, Zhejiang, China). The cytological diagnosis was made using The Bethesda System (TBS) classification criteria (TBS 2001 Revised). The cytological diagnosis was made using The Bethesda System (TBS) classification criteria (TBS 2001 Revised). The TBS classification included the following: 1. Negative, no intraepithelial lesion cells and malignant cells (NILM); 2. Abnormal epithelial cells: (1) Atypical squamous cells (ASC), including squamous epithelial cells of undetermined significance (ASC-US) and atypical squamous epithelial cells in which HSIL cannot be excluded (ASC-H),squamous low-grade intraepithelial lesions (LSIL), HSIL, and squamous cell carcinoma (SCC); (2) Abnormal glandular epithelial cells: atypical glandular cells (AGC), adenocarcinoma *in situ* (AIS), and adenocarcinoma; and (3) other malignant neoplasms.

#### Colposcopy Indications

Those with normal cytology but positive for HPV16 and HPV18 or those with HPV52 and HPV58 infection for more than 6 months with 2 consecutive positive tests will most likely need to have a colposcopy.

#### Colposcopy Methods

The standard procedure for colposcopy is used. A full colposcopic assessment of the cervical transformation zone is performed, and a multipoint biopsy plus endocervical curettage (ECC), if necessary, is performed on suspicious areas. For those without significant colposcopic abnormalities but at high risk of precancerous lesions or invasive cancer, a routine 4-point random biopsy of the cervix at 3, 6, 9, and 12 points plus endocervical curettage, if necessary, is performed. All tissues taken were sent separately for histological examination, and the pathological diagnosis was the gold standard for evaluation. The classification criteria for evaluation include the following: (1) normal or inflammatory; (2) LSIL; (3) HSIL; and (4) cervical cancer, including cervical invasive squamous carcinoma and adenocarcinoma. The colposcopic biopsy pathology results were the gold standard for statistical analysis.

#### Statistical Processing

SPSS22.0 software was used for the statistical data analysis. Statistics data are presented as frequencies and proportions. The Chi-square or Fisher’s exact test was used for the comparison of proportions between groups. A Spearman correlation was used for statistical and correlation analyses, and differences were considered statistically significant at *p* < 0.05.

## Results

### General Information on the Various HPV Subtype Groups

Of the 3,014 patients, the highest proportion of patients referred for colposcopy had HPV16 infection, followed by HPV52 and then HPV58 and HPV18. There were no statistical differences between the age groups.

### Distribution of HPV Subtypes in Different Cervical Lesions

As shown in **Table 1**, of the 3,014 patients, 78.04% were suggestive of HPV16/18/52/58 infection. As shown in **Table 2**, in HSIL and cervical cancer, the most common was HPV16, followed by HPV58 and then HPV52 (*p* < 0.05). Among normal and LSIL, HPV16 was relatively common with HPV52, followed by HPV18 and HPV58, but no statistically significant difference (*p* > 0.05) was observed.

**Table 1:** Age distribution of HPV subtype groups.

Group	Age	Number	Percentage (%)
**HPV16**	40.56 ± 10.60	883	29.30
**HPV18**	41.32 ± 9.70	419	13.90
**HPV52**	42.01 ± 10.42	588	19.51
**HPV58**	42.56 ± 10.65	462	15.33
**Others**	41.46 ± 10.44	662	21.96
**Total**	41.38 ± 10.67	3,014	100.00

**Table 2:** Distribution of HPV subtypes in different cervical lesions.

Pathology	Normal and LSIL	HSIL and Ca
**Type (%)**		
**HPV16**	420 (19.36)	325 (38.46)
**HPV18**	284 (13.09)	51 (6.04)
**HPV52**	396 (18.26)	93 (11.00)
**HPV58**	261 (12.04)	128 (15.15)
**Other**	808 (37.25)	248 (29.35)
**Total**	2,169 (100)	845 (100)

### HPV Infection in the Population

**Figure 1:**
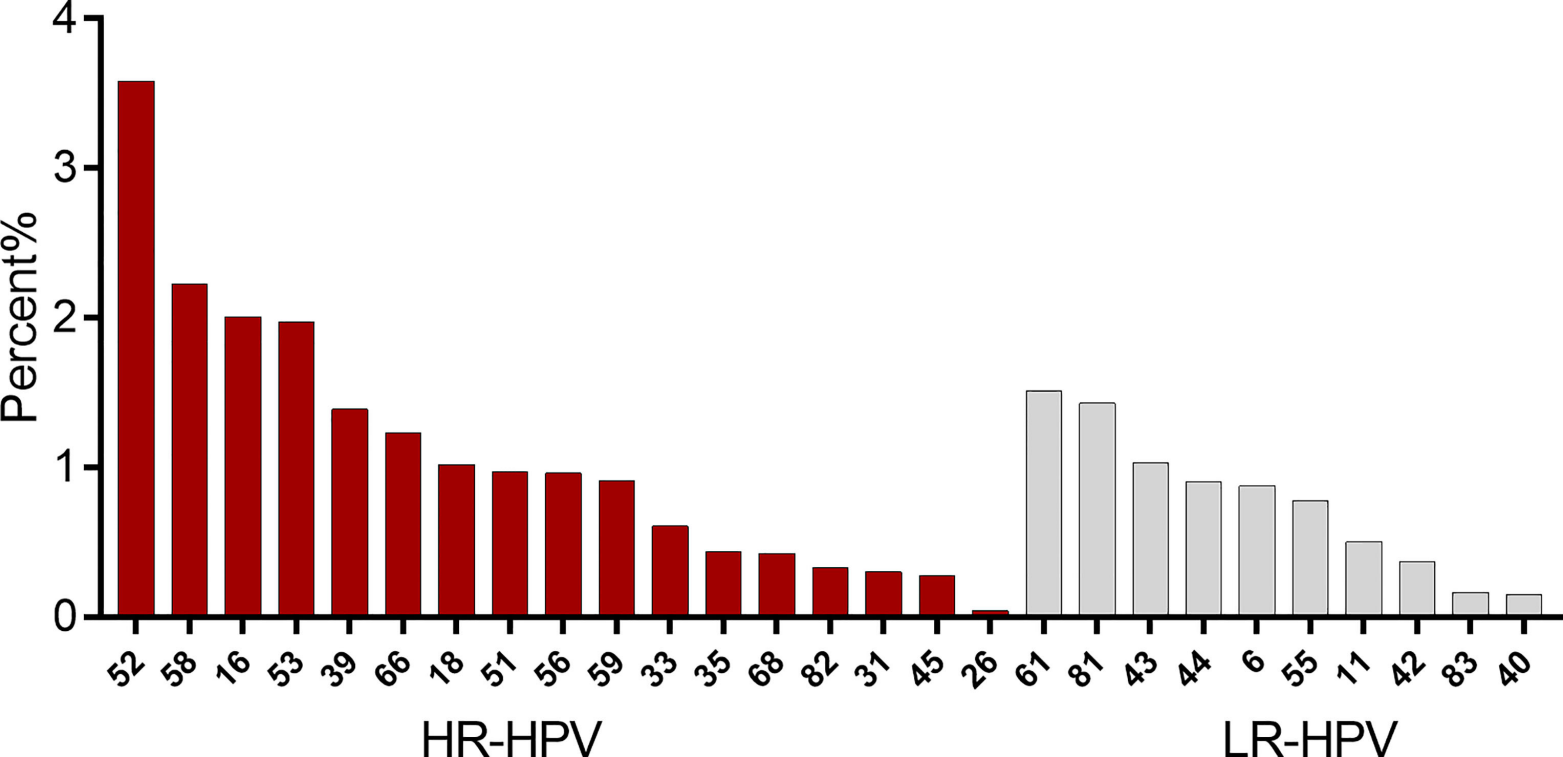
Distribution of the total infection rate (%) for the 27 HPV subtypes (left, high-risk subtypes; right, low-risk subtypes).

As shown in **Figure 1**, the most common HR-HPV genotype detected among the 244,218 patients was HPV52 (3.58%), followed by HPV58 (2.23%), HPV16 (2.01%), and HPV18 (1.02%) further down the list at 7th.

### Patient’s Colposcopic Biopsy Results in the 1,616 Patients

The previous data showed that HPV52 and HPV58 had a high prevalence of infection in the population (**Figure 1**) and were second only to HPV16 in the proportion of lesions of HSIL and cervical cancer (**Table 1**). To further explore the role of HPV52 and HPV58 in cervical disease, we analyzed 1,616 patients and identified various pathological findings, with most patients having normal or LSIL. The percentage of pathological findings were high-grade and cervical cancer: HPV16 positive (21.81%) > HPV58 positive (10.71%) (Chi-square: 31.957, *p* < 0.05), HPV58 positive (10.71%) > HPV18 positive (5.96%) (Chi-square: 4.456, *p* < 0.05), and HPV18 positive (5.96%) > HPV52 positive (5.17%) (Chi-square: 0.241, *p* = 0.624) (**Table 3**).

**Table 3:** Colposcopic biopsy results by HPV typing (*n* (%)).

	HPV16	HPV18	HPV52	HPV58	Total	*χ (* 2)
Normal and LSIL	294 (78.19)	268 (94.04)	587 (94.83)	300 (89.29)	1,449	
HSIL and cancer	82 (21.81)	17 (5.96)	32 (5.17)	36 (10.71)	167	77.2^*^
Total	376 (100)	285 (100)	619 (100)	336 (100)	1616	

Comparison of HSIL and cervical cancer in each group. ^*^p <0.001.

### Detection of High-Grade Lesions and Cancers of the Vulva and Vagina in the 1,616 Cases

As shown in **Table 4**, among the 167 patients with high-grade lesions and cancer, no high-grade vaginal or vulvar lesions developed in the HPV18-positive patients (0%), 10 cases in the 82 HPV16-positive patients (2.66%), 8 cases in the 36 HPV58-positive patients (2.38%), and 4 cases in the 32 HPV52-positive patients (0.65%). The most common was HPV16 positivity, followed by HPV58 positivity (Chi-square: 38.27, *p* = 0.003).

**Table 4:** Distribution of high-grade lesions and cancer (*n* (%)).

Types	HPV16	HPV18	HPV52	HPV58	Total	*χ (* 2)
Group						
Vulvar vagina	10 (2.66)	0 (0)	4 (0.65)	8 (2.38)	22 (1.36)	38.27^*^
Cervical	72 (19.15)	17 (5.96)	28 (4.52)	28 (8.33)	145 (8.97)	
Total	82 (21.81)	17 (5.96)	32 (5.17)	36 (10.71)	167 (10.33)	

Comparison of high-grade lesions and cancers of the vulva and vagina in each group. ^*^p < 0.05.

## Discussion

The occurrence of cervical cancer or precancerous lesions is closely linked to persistent infection with high-risk HPV types, and according to the World Health Organization, there are nearly 500,000 new cases of cervical cancer each year, of which more than 100,000 occur in China, home to about one-third of the world’s population (5).

In our data analysis, the top 3 HPV infections in the population were HPV52, HPV58, and HPV16, indicating that HPV52 and 58 infections are more common in the population. The relatively high detection rate of HPV52 and HPV58 among pathological findings suggesting high-grade cervical lesions and cervical cancer again indicates the presence of a higher rate of HPV52 and HPV58 infection in high-grade cervical lesions in addition to HPV16 and HPV18 types (7). In addition, studies have shown a relatively high prevalence of HPV58 and HPV52 in invasive cervical cancer (ICC) in Asia (8). The HPV nine-valent vaccine (HPV16/18/31/33/45/52/58/6/11), therefore, offers more protection against HPV than the quadrivalent HPV vaccine (HPV16/18/6/11) while also being safer and more cost-effective (9); this may be important to the inclusion of HPV52/58 in the HPV nine-valent vaccine. Although expanded HPV vaccination may reduce the incidence of cervical cancer, HPV vaccination rates remain low in developing countries (10, 11) due to factors such as age, education, and cost; thus, the development of a new HPV16/18/52/58 quadrivalent vaccine may help to increase vaccination rates and reduce the incidence of cervical cancer.

A meta-analysis showed that the prevalence of HR-HPV infection in women with normal cervical fluid-based cytology was 11.7% (12). In this study, we analyzed 1,616 patients who were positive for HPV16/18/52/58 alone with TCT normal, with pathological findings suggestive of normal and inflammatory conditions accounting for 64.89%–78.51%, in line with a TCT specificity of 58%–76% (13). HPV screening can compensate for the lack of liquid-based cytology and can be combined with liquid-based cytology to improve sensitivity (14).The pathological findings suggest that HPV58-positive infection is more prevalent in patients with high-grade and above lesions than HPV52 positive and also higher than HPV18 positive. This is consistent with the Kaidar–Person study, which found that HPV58 has a 1.8-fold higher association with invasive cervical cancer than HPV52 (15). We can consider HPV type 58 an important pathogen, especially in Asia (16). Therefore, in patients who are positive for HPV type 58, especially those with persistent infection despite normal TCT results, attention needs to be paid and the indications for colposcopy may be relaxed as appropriate.

There is no screening strategy for vulvovaginal high-grade lesions and cancer, given that many vaginal (17) and vulvar (18) cancers are the result of HPV infection. HPV testing can be used as a primary screening tool for the disease, and vaccination may be the only effective means of prevention (18). In this study, the detection rate of high-grade and above vulvovaginal lesions was as high as 2.66%, suggesting the need for concomitant biopsies to avoid missing lesions when we suspect vaginal or vulvar lesions. In particular, persistent infection with high-risk subtypes other than HPV types 16 and 18, such as HPV types 52 and 58, cannot exclude the possibility of vulvovaginal disease despite a normal TCT test result.

There is a limitation in this study. According to the guideline, patients with HPV16/18 infection are immediately referred for colposcopy, but patients with HPV52/58 infection are not all referred for colposcopy and tissue biopsy if TCT test results are normal. The sample included in this study is only a portion of the overall total number of samples. Thus, there is bias here. We will further expand the sample size.

## Conclusion

1. In patients with positive HPV58 alone and normal TCT, the indications for colposcopy may be relaxed, with particular attention paid to the possibility of vulvar and vaginal lesions.
2. Patients with a positive HPV type 52 alone and normal TCT may be considered for a follow-up review and, if necessary, a colposcopy.
3. The development of a more suitable HPV vaccine for the Asian population, such as HPV16/18/52/58, may better protect women’s health.

## Data Availability Statement

The original contributions presented in the study are included in the article/supplementary material. Further inquiries can be directed to the corresponding authors.

## Author Contributions

BY proposed the research object, collected the data, analyzed the data, and wrote the article. QX has contributed equally to this Work, is considered as co-first author. ZZ collected and analyzed the data. JZ, YX, and LH collected the data. YH proposed the research object and analyzed the data. QT proposed the research, collected and analyzed the data, is considered as co-corresponding author. JC collected, analyzed, wrote portion the manuscript and found the right journal, is the corresponding author of this paper. All authors listed have made a substantial, direct, and intellectual contribution to the work.

## Funding

This research was partially supported by Wenzhou Science and Technology Bureau Project–Y20190417.

## Conflict of Interest

The authors declare that the research was conducted in the absence of any commercial or financial relationships that could be construed as a potential conflict of interest.

## Publisher’s Note

All claims expressed in this article are solely those of the authors and do not necessarily represent those of their affiliated organizations, or those of the publisher, the editors and the reviewers. Any product that may be evaluated in this article, or claim that may be made by its manufacturer, is not guaranteed or endorsed by the publisher.
